# Passive Microwave Radiometry for the Diagnosis of Coronavirus Disease 2019 Lung Complications in Kyrgyzstan

**DOI:** 10.3390/diagnostics11020259

**Published:** 2021-02-07

**Authors:** Batyr Osmonov, Lev Ovchinnikov, Christopher Galazis, Berik Emilov, Mustafa Karaibragimov, Meder Seitov, Sergey Vesnin, Alexander Losev, Vladislav Levshinskii, Illarion Popov, Chingiz Mustafin, Turat Kasymbekov, Igor Goryanin

**Affiliations:** 1 Educational-Scientific Medical Center, Kyrgyz State Medical Academy (KSMA), 720040 Bishkek, Kyrgyzstan; z_cool@mail.ru; 2 Medical Microwave Radiometry LTD, Edinburgh EH10 5LZ, UK; ksigne@yandex.ru (L.O.); vesnin47@gmail.com (S.V.); 3 School of Informatics, University of Edinburgh, Edinburgh EH8 9AZ, UK; chrisgalazis@gmail.com; 4 Internal Diseases Department, International Medical University (IMU), 720021 Bishkek, Kyrgyzstan; emilov9090@mail.ru (B.E.); dr.korkunov@gmail.com (M.K.); seiitov@outlook.com (M.S.); 5 RTM Diagnostic, LLC, 55/59 b.1, B. Pochtovaya, 105082 Moscow, Russia; 6 Faculty of Mathematics and Information Technology, Volgograd State University, 400062 Volgograd, Russia; allosev59@gmail.com (A.L.); vladi.lev.email@gmail.com (V.L.); popov.larion@yandex.ru (I.P.); 7 Komfort Clinic, 720020 Bishkek, Kyrgyzstan; chingis.x@gmail.com (C.M.); medcenter-kg@mail.ru (T.K.); 8 Biological Systems Unit, Okinawa Institute Science and Technology, Kunigami District, Okinawa 904-0495, Japan

**Keywords:** COVID-19, passive microwave radiometry (MWR), infrared radiometry (IR), RT-PCR, CT

## Abstract

The global spread of severe acute respiratory syndrome coronavirus 2, which causes coronavirus disease 2019 (COVID-19), could be due to limited access to diagnostic tests and equipment. Currently, most diagnoses use the reverse transcription polymerase chain reaction (RT-PCR) and chest computed tomography (CT). However, challenges exist with CT use due to infection control, lack of CT availability in low- and middle-income countries, and low RT-PCR sensitivity. Passive microwave radiometry (MWR), a cheap, non-radioactive, and portable technology, has been used for cancer and other diseases’ diagnoses. Here, we tested MWR use first time for the early diagnosis of pulmonary COVID-19 complications in a cross-sectional controlled trial in order to evaluate MWR use in hospitalized patients with COVID-19 pneumonia and healthy individuals. We measured the skin and internal temperature using 30 points identified on the body, for both lungs. Pneumonia and lung damage were diagnosed by both CT scan and doctors’ diagnoses (pneumonia+/pneumonia−). COVID-19 was determined by RT-PCR (covid+/covid−). The best MWR results were obtained for the pneumonia−/covid− and pneumonia+/covid+ groups. The study suggests that MWR could be used for diagnosing pneumonia in COVID-19 patients. Since MWR is inexpensive, its use will ease the financial burden for both patients and countries. Clinical Trial Number: NCT04568525.

## 1. Introduction

A significant number of deaths occur in coronavirus disease 2019 (COVID-19) patients with multiple concomitant diseases around the world, such as interstitial pneumonia, acute respiratory distress syndrome, and subsequent multiple organ failure [1]. Severe lung damage has been reported at any age. In infected persons, various degrees of pneumonia and radiological signs are observed [2,3]. Currently, most research are focused on the use of computed tomography (CT) to identify the chest manifestations of COVID-19 [4,5]. In contrast to the high chest CT sensitivity, the specificity is relatively low (approximately 25–33%). There are obstacles to using CT due to the need for infection control related to patient transportation, need for disinfection of CT rooms after patient examination, and the lack of accessibility to the CT equipment [6]. In the early stage of disease progression, ground-glass opacities are the predominant lesions. In the next stage, crazy paving patterns highlights the inflammatory changes. The peak stage is marked by fibrosis and diffuse damage. These CT lesions are also observed in other pneumonia and non-infectious inflammatory lung diseases, but in a pandemic context, CT shows diagnostic potential for COVID-19, especially for patient triage [7,8,9].

In healthcare settings with limited polymerase chain reaction (PCR) capacity and long turnaround times, chest CT is proposed as an alternative for COVID-19 diagnosis. Studies supporting chest CT as a first-line diagnostic tool for COVID-19 have revealed several methodological concerns [10,11,12]. There are associated costs and procedural risks with CT [13,14,15].

The other method for diagnosing COVID-19, reverse transcription PCR (RT-PCR), has a variable sensitivity as low as 70% [16]. The specificity of viral swabs in clinical practice varies depending on the site and quality of sampling. In one study, the RT-PCR sensitivity in 205 patients varied, at 93% for bronchoalveolar lavage, 72% for sputum, 63% for nasal swabs, and only 32% for throat swabs [17]. The test results are also likely to vary depending on the stage and degree of viral load or clearance [17,18]. The use of repeat RT-PCR testing as a standard is likely to address the probably low specificity as well as the true rate of false negatives, because not all patients receive repeat testing results. Therefore, another safer and inexpensive method is required to replace and/or compliment CT and PCR tests.

Microwave radiometry (MWR) technology [19] has already been used for the early diagnosis of cancer and other diseases. It measures emission in the microwave range of tissues/organs to a depth of 5 cm beneath the skin. Increased microwave emission could result from inflammation, while decreased emission could be due to fibrosis. The advantage of MWR is that the temperature manifestations can be revealed before any structural changes can be registered.

During the 1980s–1990s, there were several studies on the identification of excess microwave emission due to fluid in the lungs (on phantoms), which could be an indication of inflammatory processes, pneumonia, cancer, and other lung disorders [20,21]. Later, clinical studies confirmed the results for lung cancer [22,23].

The purpose of this study was to investigate the value of MWR and compare microwave (internal temperature) and infrared emissions (skin temperature) from the left and right lungs with those of chest CT and RT-PCR to determine the diagnostic performance of MWR in individuals with COVID-19 symptoms.

## 2. Materials and Methods

**Figure 1:**
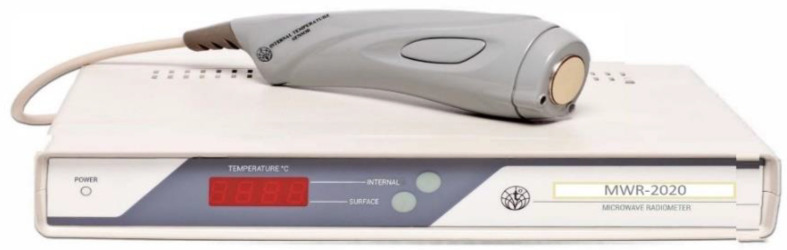
MWR2020 (former RTM-01-RES).

In this trial (Kyrgyz Committee Clinical Trial Number: 01-2/141; 27 May 2020), from 1 June 2020 to 1 August 2020, we performed parallel MWR, PCR, and CT tests for individuals with COVID-19 admitted to the hospital for medical emergencies related to COVID-19 with suspected pneumonia. CT scans were performed with a Siemens Ecoline CT scanner (Munich, Germany) and as described in a standard routine by a hospital physician who was not directly involved in this research. RT-PCR was performed using “DNA technology” kits (DNA-Technology LLC, Moscow, Russia). For the MWR and infrared (IR) measurements, MWR2020 (RTM-01-RES) (Figure 1) was used (MMWR LTD, Edinburgh, UK). It is a unique commercial CE-marked device registered in the UK MHRA MDN 40802 (Freepost Yellow Card scheme-MHRA, London, UK) as a microwave thermography system for clinical studies. The device is already registered in Kyrgyzstan for breast cancer diagnosis. The technical parameters are in Table 1.

This is an analysis of a single-center prospective trial on consecutive individuals admitted to the Medical Center of Kyrgyz State Medical Academy (KSMA) BICARD clinic from 1 July 2020 to 1 August 2020. KSMA is a central-network regional hospital that provides tertiary healthcare for a community of 500,000 inhabitants. All individuals admitted to the hospital on the clinical suspicion of COVID-19 pneumonia (confirmed by experienced pulmonologists, so that symptomatic individuals were the inclusion criteria) received a combined screening using chest CT and RT-PCR. We used COVID-19 case definition as specified in the World Health Organization (WHO) document [24] for classifying symptomatic individuals. Patients with lung comorbidity and individuals without COVID-19 symptoms did not receive chest CT. Exclusion criteria included lung comorbidity (such as exacerbation of chronic obstructive pulmonary disease (COPD) and very severe COPD with hypoxia (FEV1 < 40%, saturation < 92% at an altitude of 760 m)), comorbidities (such as cardiovascular diseases, including unstable systemic arterial hypertension, coronary heart disease, stroke, sleep apnea, pneumothorax lasting past two months, neurological, rheumatological, or psychiatric illnesses), excessive smoking (>20 cigarettes per day), and kidney failure.

The study NCT04568525 was approved on 27.02.2020 by the Kyrgyz Republic Review Board, and informed consent was obtained from all the subjects. Overall, we measured internal (MWR) and skin (IR) temperatures in 208 subjects (84 males and 124 females) aged 18 to 75 years old. A total of 135 patients were hospitalized with pneumonia symptoms in KSMA Medical Center and the BICARD clinic, Bishkek, while 73 subjects were selected from healthy volunteers with no COVID-19 and pneumonia symptoms. RT-PCR was performed for each hospitalized subject, and 112 subjects were found to be positive for COVID-19 using RT-PCR test.

Healthy volunteers with no COVID-19 and pneumonia symptoms did not receive chest CT, but they were tested using MWR. Body mass index (BMI), auxiliary (armpit) temperature, and SpO_2_ were additionally assessed as part of the hospital admission routines.

**Figure 2:**
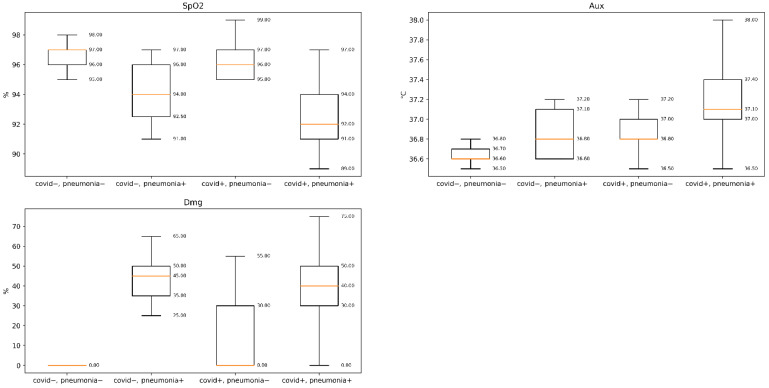
Box plot distribution of the clinical parameters. **SpO2** is blood oxygen saturation, **Aux** is auxiliary (armpit) temperature, **Dmg** is overall ercentage of lung damage assesment based on CT scan.

**Figure 3:**
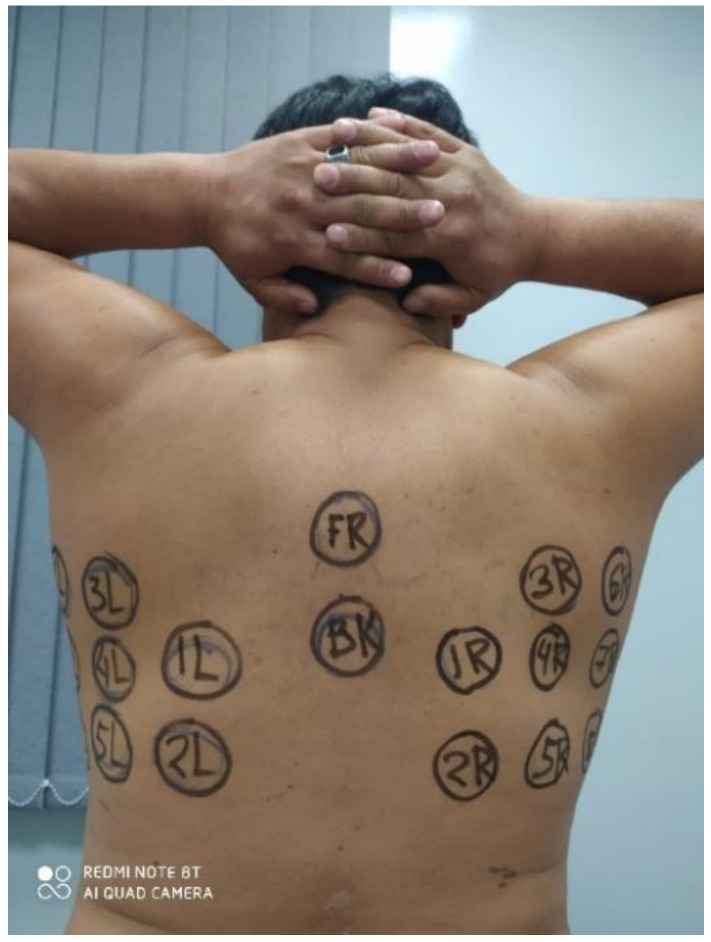
Measurement points.

**Figure 4:**
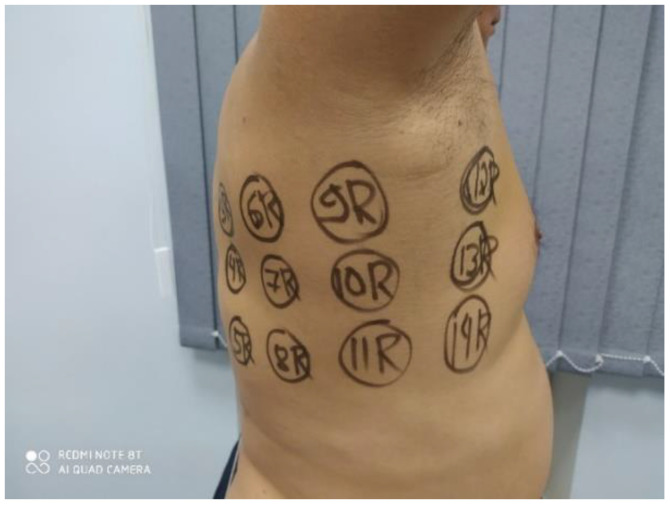
Measurement points.

As there were no previous measurements of the lungs using MWR, a new measurement technique was designed for this research. Thirty points were identified on the body, 28 symmetrical (R1-R14, –L1-L14) and 2 controls (T1 and T2), as shown in Figure 2, Figure 3 and Figure 4. The software application “RTM Diagnosis 1.79” was configured to perform these measurements, record the temperatures, and plot the corresponding temperature fields. The measurement of a single point takes no more than 10 s, and thus the overall measurement cycle is approximately 5 min. The existing measurement technique, for example, for the breast, requires a cloth-off condition. However, it is not very practical and leads to patient discomfort. To assess the possibility of measurements with the clothes on, we measured 83 subjects through thin clothes and 125 with the clothes off.

### Statistical Approach

A total of 30 internal (MWR) temperatures and 30 skin (IR) temperatures were measured, with a total of 60 values per subject, and a total of 208 × 60 = 12,480 values overall.

As a reference, 2 conditions were separately used to confirm the diagnosis of pneumonia on CT (pneumonia+, pneumonia−) and positive RT-PCR test (covid+, covid−). Thus, there were 4 groups: (covid+/pneumonia+), (covid−/pneumonia−), (covid+/pneumonia−), and (covid−/pneumonia+), and statistical and Deep Neural Network analyses were aimed to determine whether MWR could predict the possibility of the subject being in a specific group (Table 2).

## 3. Results

### 3.1. Clinical Results

RT-PCR test was performed for each hospitalized subject, and 112 subjects were found to be positive. On the basis of the CT scans, we found that 122 patients were diagnosed with pneumonia. Pneumonia (CT) and RT-PCR results were used as reference. Ambient temperature varied from 27 to 30 °C, which is slightly higher than the recommended temperature (20–24 °C). Recently, we have shown that the device can be used at high room temperatures [25,26]. The average BMI for all subjects was 26.3 within 24 h of admission; all individuals were imaged by CT. Radiologists >5 years of experience reviewed the CT test and determined the left and right lung damage percentage (denoted *Dmg*). They were blinded to (a) symptomatic status and RT-PCR results. Median lung damage percentage for subjects with positive RT-PCR results was 40%, and median axillary temperatures were 36.6 °C and 36.7 °C for the covid− and covid+ groups, respectively. Distributions of the parameters within groups are depicted in Figure 2.

**Figure 5:**
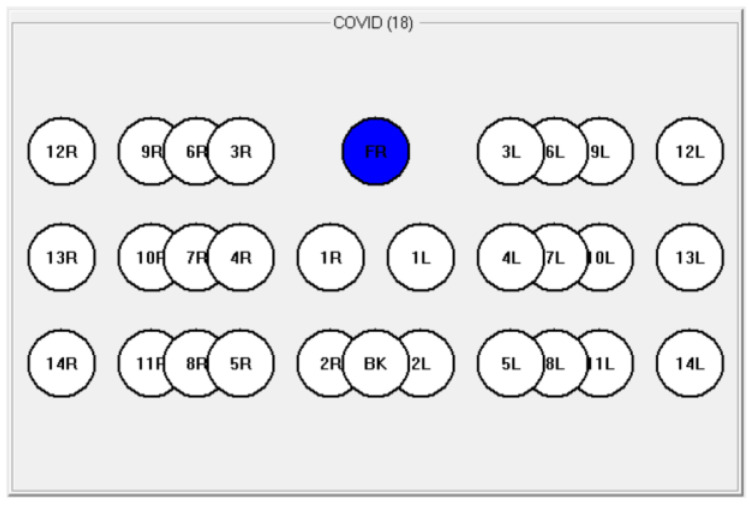
The measurement scheme in the software “RTM-Diagnosis”.

MWR temperature measurements were performed in the lung projections, as shown in Figure 3, Figure 4 and Figure 5, in 30 symmetrical points pairwise positions. All data are shown in the Supplementary material Table S1.

### 3.2. Clinical Images

**Figure 6:**
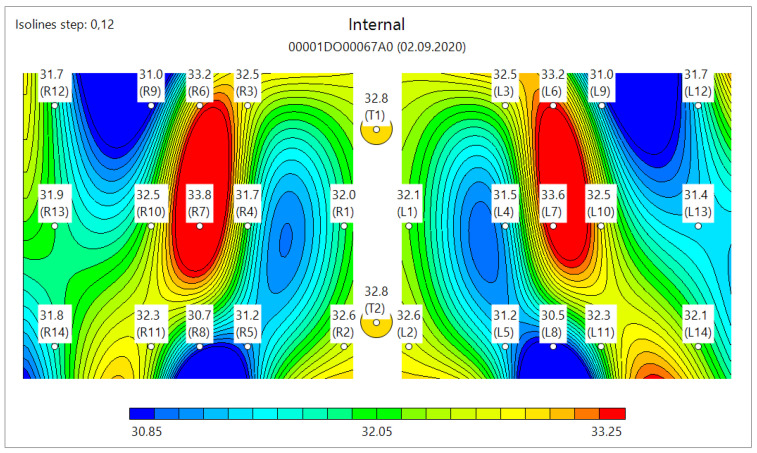
Typical microwave image of coronavirus disease 2019 (COVID-19) in the left and right lungs. Large internal temperature difference is shown in blue (low temperature, due to fibrosis) and red (high temperature, due to inflammation).

**Figure 7:**
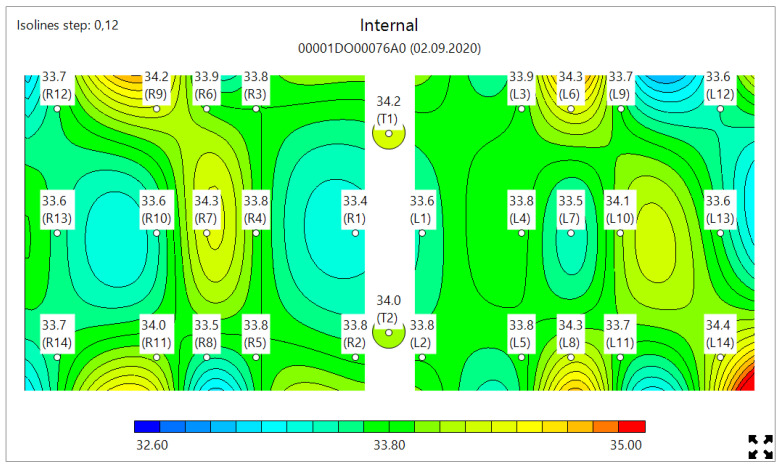
Typical microwave image of the healthy lungs showing no blue or red areas.

**Figure 8:**
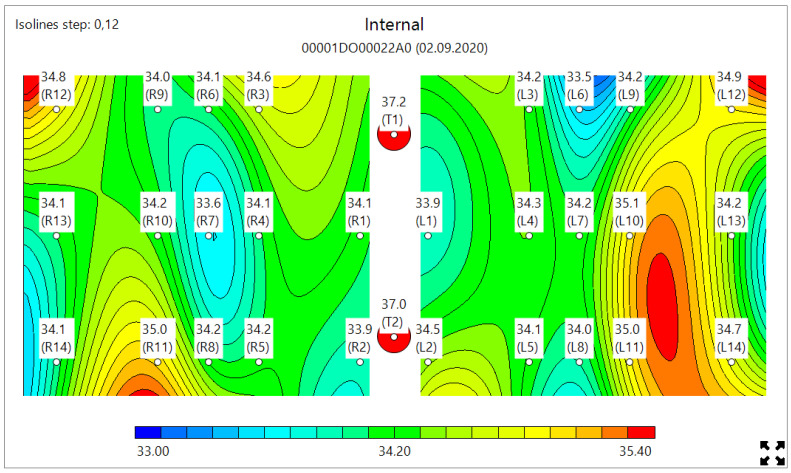
Typical microwave image of non-COVID-19 pneumonia showing red (inflammation area only in the left lung) and no blue areas (due to fibrosis in both lungs).

Figure 6 shows the typical MWR image of COVID-19 pneumonia. On both the left and right lungs, there are a large internal temperature difference in the blue areas (low temperature, due to fibrosis) and red areas (high temperature, due to inflammation). Figure 7 shows the lungs of healthy individuals, while Figure 8 depicts no COVID-19 pneumonia, where only the regions of inflammations could be observed, but no blue zones are visible.

### 3.3. Statistical Results

We measured four temperature values: $T_{\mathrm{int},R},\;T_{\mathrm{int},L},\;T_{\mathrm{sk},R},\;T_{\mathrm{sk},L}$ at 14 symmetrical points on the body (right, left) and two asymmetrical points.

From existing microwave diagnostic practice, point temperature values are usually not informative due to individual human tissue variance; however, patterns are being formed.

Therefore, we hypothesized that

1. The difference in symmetrical points should be less than 0.5 °C in healthy patients and be of greater value in patients with pneumonia;
2. The difference between internal and skin temperatures should be uniform in healthy patients but might show local abnormalities in patients with pneumonia;
3. The difference between the hottest and coldest points should be less than 2 °C in healthy patients.

We introduced 16 aggregated metrics, each of which was calculated pointwise. The median was preferred over a simple mean intentionally in order to reduce the effect of poorly performed measurements.

- **Asymmetry** (AS) expresses the difference between symmetrical positions, and was calculated as follows:
  $$\left|T_{int,R}-T_{int,L}\right|+\left|T_{sk,R}-T_{sk,L}\right|$$
- **Median asymmetry** (#1) shows the overall disbalance between the left and right sides; the standard deviation of asymmetry (#5) aggregates the local irregularities, while the maximum asymmetry highlights the asymmetry at a single point (#9).
- **Asymmetry inverse** (ASIN) (#2) reveals the condition when the internal and skin temperatures show asymmetry with significantly different magnitudes or signs.
  $$\left|\left(T_{int,R}-T_{int,L}\right)-\left(T_{sk,R}-T_{sk,L}\right)\right|$$

ASIN shows the overall equality between internal and skin asymmetry; ASIN max (#10) shows the irregularity at a single point.

- **Spread** (SP) expresses the difference between the internal and skin temperatures.
  $$T_{int,R}-T_{sk,R}+T_{int,L}-T_{sk,L}$$
- **SP median** (#3) shows the overall change, which is caused by the systematic changes in the metabolism or thermal properties; SP std (#7) shows the non-uniformity of the internal-skin difference; SP max (#11) highlights the anomaly at a single point.
- **Relative increase** (RI) aimed to reveal the irregular points of increase or decrease. It is calculated as the value of the difference between the internal value and the median of all internal values for that patient
  $$\{\begin{array}{c} T_{int,R}-med\left(T_{int}\right),\;if\;\left|T_{int,R}-med\left(T_{int}\right)\right|>\left|T_{int,L}-med\left(T_{int}\right)\right|\;\\ T_{int,L}-med\left(T_{int}\right),otherwise \end{array}$$

RI median (#4) indicates the overall tendency of increase or decrease over the baseline uniform distribution, and RI max (#12) shows the hottest position.

- **Internal median** (#13) shows a shift in the baseline level of the internal temperature.
- **Internal percentile interval** (#14) (5–95%) is aimed at measuring the spread between the hottest and coldest measured points. The same applies for the skin (#15, #16).

Each metric was separately tested to show statistically significant differences between the four groups: (covid+/pneumonia+), (covid−/pneumonia−), (covid+/pneumonia−), and (covid−/pneumonia+).

Then, we used the one-way multi-group analysis of variance (ANOVA) (Table 3) to check for similarities in means between the four groups. For metrics with *p* < 0.05, pairwise Tukey test (Table 3) was performed to assess differences in metrics between groups’ pairwise. Pairs are meant to be significant if *p* < 0.05 in Tukey test. These assessments were performed using Python script [27,28,29].

The pairwise Tukey test (Table 4) shows that the border groups (covid−/pneumonia−) and (covid+/pneumonia+) differed in almost all the relevant features. COVID-19 patients were characterized by an increase in internal temperature (+1.3 °C) and decrease in skin temperature (−0.6 °C), and consequently, increase in the spread between the internal and skin (median from +1.48 °C, up to +2.7 °C at single points).

The analysis showed that groups measured with clothes off had more pronounced differences than those measured with clothes on. For the groups with clothes on, covid+/pneumonia+ patients had their median skin temperatures lowered by 1.0 °C with identical internal temperatures. It is important to combine the internal and skin temperatures to estimate and determine the significant differences between the four groups. We originally hypothesized that absolute temperature values will be least informative, but we found that the greatest effect was a decrease in the absolute value of the skin temperature and an increase in the spread (SP). Asymmetries showed no change. This might be explained mostly by the bilateral cases of COVID-19 pneumonia.

On the basis of the statistical results, for subsequent AI analysis, we decided to combine the clothes on/off groups together and train the network using the most informative aggregated metrics along with the raw temperature data.

### 3.4. Deep Neural Network Results

We used as a starting model the same deep neural network (DNN) we have earlier applied for breast cancer diagnostics [30,31,32]. For this iteration, we further improved the dense model by incorporating characteristics from the Cascade Correlation Neural Network (CCNN). This includes the addition of skip connections and an increased number of hidden layers in the model. Each layer has as input the original input points and the output of all previous layers, which is aggregated by concatenation. Additionally, regularization techniques have been included to avoid early overfitting, such as random node dropout, Gaussian noise, and L1/L2 weight regularizers.

Thus, it allows the model to identify both shallow and deep metrics in the training set. In addition, it overcomes some of the limitations of the previous CCNN model. Training of the CCNN was significantly slower depending on the number of candidates per iteration (model size is not a constraint for this problem with current hardware). Furthermore, having a smaller number of nodes per layer constrains the breadth of features that can be extracted. Finally, with the step-by-step weight freezing, it limits the identification of more complex features as these can no longer influence shallower ones.

The problem, from the perspective of the network, is being treated as a multi-label and multi-output task. For each of the potential labels of pneumonia (−/+) and COVID-19 (−/+), a binary output node is added to the network. The loss function used to minimize for both cases is the binary cross entropy, in which the global goal is to minimize the sum of the two. During training, two sets of weights are stored, those that result in the minimum loss value individually for the labels. Following for the inference phase, the respective weights are loaded to make the predictions.

The data were split to train (60%), validation (20%), and test (20%) sets. The distribution of cases of pneumonia and COVID-19 were balanced across the three datasets. Additionally, for the training set, the data were expanded by switching the symmetrical points of the left lung with the right and vice versa. A total of five experiments were conducted with the available data, raw temperature, raw temperature, and metadata; all aggregated pointwise metrics (AS, ASIN, SP, RI, Int, and Sk), raw temperature, and pointwise metrics; and, finally, raw temperature, pointwise metrics, and metadata. After the model is trained, the receiver operating characteristic is used with the validation data to determine an optimal threshold for each output to balance the true and false positive rates.

The results of the experiments are summarized in Table 5. The overall best performance was achieved when using the raw temperature readings and the aggregated pointwise metrics. It achieved a sensitivity of 71.05% and specificity of 74.35%. The highest sensitivity achieved, of 79.85%, was with using the temperature readings in conjunction with the metadata. However, it obtained a sensitivity of 48.37%. In contrast, the highest specificity, of 77.29%, was obtained using only the aggregated metrics. The sensitivity fell at 50.99%.

We conducted a second iteration of experiments that were based on the raw temperature reading and the extracted metrics, as they were the best results thus far. Knowing that whether a patient is wearing clothes or not during the recording can affect the results, we prepared a second batch of experiments. While including all the metadata information had a negative impact, we reran our network with adding only the clothes flag. Additionally, we experimented with creating an ensemble model. One network was trained only with data the patients were wearing their clothes and the other that they were not.

The results of the second set of experiments are summarized in Table 6. Including the flag helped to improve the results. The sensitivity increased from 71.05% to 76.47%. With a smaller increase for the specificity, it changed from 74.35% to 76.47%. The ensemble approach significantly reduced the specificity to 47.06%. This can be attributed to the fact that each network was now trained on a smaller set of data. Specifically, there were more similarities than dissimilarities between patients wearing or not wearing clothes.

In general, the model had slightly better performance in correctly detecting presence of pneumonia in comparison to COVID-19. However, more data will be required to better capture the diversity and complexity of the cases and allow the model to effectively generalize. This also extends to when the patient metadata/additional information is included, in which not all information offers benefits to the classification. One value that has a positive impact is the clothes flag as it directly contributes to temperature differences captured at the skin.

## 4. Discussion

We aimed to investigate the performance of MWR to diagnose theCOVID-19 lung complications. Most studies used CT results as positive or negative, often without a clear definition of a positive CT. One large study reported a 97% sensitivity of chest CT for COVID-19 diagnosis but with a poor specificity of 25% [32]. The actual clinical value of a positive CT result to confirm, or negative test results, strongly depend on disease progression [33]. In recent trials, the sensitivity of chest CT was insufficient to exclude severe acute respiratory syndrome coronavirus 2 (SARS-CoV-2) infection, which supports the consensus statement that chest CT should not be used as a diagnostic test alone [34]. IR scanners cannot measure the internal temperature of organs, only the skin temperature can.

MWR aggregate metrics had good diagnostic performance for COVID-19 pneumonia but could not replace RT-PCR as a diagnostic test. Therefore, we suggest using MWR as a complementary tool in individuals with COVID-19 symptoms and for the early screening of asymptomatic infections. There are no ideal diagnostic methods. In some cases, all the symptoms show in the COVID-19 patient, but the PCR test is negative. CT images do not show lung damage or CT images may be unavailable.

There are five major observations about measured skin and internal temperature values [35,36,37], which we have confirmed in this study.

1. Individual data points are hardly informative, due to inaccuracies in measurements and noise-induced randomness.
2. The mean value has limited informativeness due to individual variations in metabolism, conductivity of the tissues, and changes in the ambient temperature.
3. An increase or a decrease in a point relative to its neighbors might be informative (so-called thermal heterogeneity).
4. An increase or a decrease in a point relative to the symmetric point on the body might be informative (so-called thermal asymmetry).
5. An increase or a decrease in the microwave temperature value relative to the infrared temperature value might be informative (so-called thermal convergence), especially compared to its neighbors.

It is also very important to consider that this methodology will be readily accessible to the Low Middle Income Countries, and that it is even more convenient to use at the primary healthcare level. The primary healthcare is the first line of treatment for patients worldwide, and they are the first point of contact for the patients. MWR could identify early stage lung damage to help the doctors in their decision-making process for patients with some COVID-19 symptoms.

Usually, COVID-19 progresses through several stages. From days 1–4 of the disease progression, fever persists, as does the cough. Second, breathing difficulty begins on day 5. It is especially likely to happen if the person has a preexisting condition or is older. The third stage is from days 6–10 when the outcome could lead to hospitalization. MWR could be used at these stages to identify high-risk patients and to adjust their therapy. MWR usually fails when the covid+ patient has no symptom at all, or when the patients have very high fever >38.5 °C. It is not practical to use MWR in intensive care units. Initial MWR training is required, but the procedure itself is not complex and is similar to the skin temperature measurements performed on different parts of the human body.

Our study showed that MWR+/MWR– lung damage predictions could be used for screening and stratifying patients into six groups:

- Covid+, pneumonia+, MWR+, accident and emergency hospitalization; usually, the patients are already hospitalized.
- Covid− or RT-PCR test not available, pneumonia+, MWR+ hospitalization; usually the patients are already hospitalized.
- Covid− or RT-PCR test not available, pneumonia− (or CT test not available,), MWR+; consultancy with a specialist, repeat or take PCR test; repeat, or take CT test and MWR examination.
- Covid+, pneumonia−, MWR−, repeat MWR test; most likely it is asymptomatic COVID-19, and no further action is required.
- Covid−, pneumonia+, MWR−, repeat PCR and MWR tests; usually, the patients are already hospitalized.
- Covid−, pneumonia−, MWR−; no further action is required.

The test could preferably be done with the clothes off, but when it is not possible, thin clothes are allowed. Further studies are being carried out by the support of the Kyrgyz Government to validate this protocol. In the follow-up study, we collected more data and training on the Deep Neural Network system to include measurable parameters from blood tests (C-reactive protein) and SpO2 in order to improve MWR diagnostics power. MWR could be used for early lung diagnosis more widely where access to CT/PCR is limited, including but not limited to

- Nursery homes;
- Ships;
- Remote locations (highlands, islands, deserts);
- Boarder security;
- Detention centers.

Our study has some limitations. It was conducted in a time frame when high rates of COVID-19 and a low prevalence of other viral pneumonia were occurring. A higher incidence of seasonal respiratory viral infections will likely decrease the specificity of MWR. Healthy individuals were underrepresented in our dataset.

All three hypotheses were fully confirmed both in statistical and clinical aspects. Hypothesis #1—according to five main signs of inflammation from the course of physiology (rubor, tumor, dolor, calor, functio laesa), almost all of them are inherent in pneumonia, whatever etiology it may be. In hypothesis #2, there is almost always a local difference. In hypothesis #3—when examining healthy people, a deviation of 2 degrees is associated with the thickness of the skin and the thickness of the clothes in which it came, and the external temperature factor also affects.

We have been actively using this technique for 4 months in clinical practice and it is of great benefit, we do not notice any obvious errors.

COVID-19 could damage the brain, heart, gut, and other organs. MWR is already being used for the diagnosis of different diseases [19]. In the future, it could be used for the full body scan, including the head (brain), wrist (cardiovascular), lung (respiratory), and guts (GI) to assess organ damage and eliminate the risk of COVID-19 rehabilitation stage.

Our newly developed wearable MWR sensor could be used to monitor patients 24/7, but further development is required [38].

## 5. Conclusions

This study suggests that MWR could be used for the diagnosis of COVID-19 pneumonia. Since MWR is inexpensive, it could ease the financial burden for both the patients and countries, especially in LMIC.

## Figures and Tables

**Table 1:** Technical specification.

Key Features (MWR2020)	
Temperature detection depth, cm	3–7
Accuracy of internal temperature measurements (microwave range), °C	0.2
Measurement time, s	8
Antenna diameter, mm	39
Accuracy of skin temperature measurements (infrared range), °C	0.2
Weight, kg	2.5
Power, W	20

**Table 2:** Group sizes.

Group	Covid−	Covid+
Pneumonia−	77	9
Pneumonia+	19	103

**Table 3:** ANOVA statistics.

	Clothes on			Clothes off		
Aggregated Metrics	f-Value	*p*-Value	Reject Null-Hyp (*p* < 0.05)	f-Value	*p*-Value	Reject Null-Hyp (*p* < 0.05)
1. AS median	0.34	0.79	No	3.21	0.03	Yes
2. ASIN median	0.05	0.98	No	3.08	0.03	Yes
3. SP median	3.65	0.02	Yes	12.35	4.18 × 10^7^	Yes
4. RI median	0.81	0.49	No	2.90	0.04	Yes
5. AS std	0.30	0.82	No	0.05	0.98	No
6. ASIN std	0.58	0.62	No	1.32	0.27	No
7. SP std	0.30	0.83	No	2.73	0.05	Yes
8. RI std	0.32	0.81	No	0.11	0.96	No
9. AS max	0.24	0.86	No	0.05	0.99	No
10. ASIN max	0.67	0.57	No	1.05	0.37	No
11. SP max	0.08	0.97	No	5.67	1.1 × 10^3^	Yes
12. RI max	0.71	0.55	No	1.12	0.34	No
13. Int median	2.54	0.06	No	2.88	0.039	Yes
14. Sk median	7.26	2.2 × 10^4^	Yes	6.09	6.7 × 10^4^	Yes
15. Int interval	3.16	0.03	Yes	0.22	0.87	No
16. Sk interval	0.93	0.42	No	0.36	0.77	No

**Table 4:** Significant pairs.

Criteria	Significant Pairs (Clothes on)	Significant Pairs (Clothes off)
SP median	Covid− pneumonia−/covid+ pneumonia+ (delta = +1.1, *p* = 0.0075)	Covid− pneumonia−/covid+ pneumonia+ (delta = +1.48, *p* < 0.001)
SP std		Covid− pneumonia−/covid+ pneumonia+ (delta = +0.40, *p* = 0.025)
SP max		Covid− pneumonia−/covid+ pneumonia+ (delta = +2.72, *p* < 0.001)
Int median		Covid− pneumonia−/covid+ pneumonia− (delta = +1.31, *p* = 0.043)
Sk median	Covid− pneumonia−/covid+ pneumonia+ (delta = −1.00, *p* = 0.001) Covid− pneumonia+/covid+ pneumonia+ (delta = −0.85, *p* = 0.008)	Covid− pneumonia−/covid+ pneumonia+ (delta = −0.64, *p* = 0.008) Covid− pneumonia+/covid+ pneumonia− (delta = +3.12, *p* = 0.006)

**Table 5:** Results from neural network on different inputs.

Experiment	Sensitivity	Specificity
Raw temperatures	71.05%	57.52%
Raw temperatures and metadata	79.85%	48.37%
Metrics	50.99%	77.29%
Raw temperatures and metrics	71.05%	74.35%
Raw temperatures, metadata, and metrics	70.95%	48.18%

**Table 6:** Results from neural network on including the clothes flag. The base input data is raw temperatures and the set of computed metrics.

Experiment	Sensitivity	Specificity
Raw temperatures and metrics	71.05%	74.35%
Raw temperatures, metrics, and clothes flag	76.19%	76.47%
Raw temperatures and metrics (ensemble clothes on/off)	76.19%	47.06%

## Data Availability

Data is contained within the article or supplementary material.

## Supplementary Materials

The following are available online at https://www.mdpi.com/2075-4418/11/2/259/s1, Table S1: The dataset under analysis.
